# New considerations for representing moisture in indoor thermal conditions: Associations between enthalpy, cognitive performance, and thermal sensations

**DOI:** 10.1016/j.indenv.2025.100098

**Published:** 2025-05-11

**Authors:** Sandra Dedesko, Joseph Pendleton, Anna S. Young, Brent A. Coull, John D. Spengler, Joseph G. Allen

**Affiliations:** a Harvard T.H. Chan School of Public Health, Boston, MA, USA; b Harvard Graduate School of Arts and Sciences, Cambridge, MA, USA; c Emory Rollins School of Public Health, Atlanta, GA, USA

**Keywords:** Thermal comfort, Productivity, Temperature, Relative humidity, Indoor environmental quality

## Abstract

Motivated by limitations with the use of temperature and thermal comfort models in relation to occupant health outcomes, this work investigates numerous characterizations of thermal conditions and associations among these thermal variables, cognitive performance, and thermal perceptions. Measurements of classroom dry-bulb temperature and relative humidity were used to calculate a suite of eleven thermal variables, which were paired with thermal sensation votes and cognitive test responses from graduate students attending classes in these monitored spaces, resulting in an analysis dataset of 273 observations from 54 participants. Results from Spearman Rank correlation coefficients, factor analysis, and principal component analysis suggest that the eleven thermal variables cluster into three groups that reflect variations in indoor temperature, indoor relative humidity, and indoor-outdoor differences. While several variables appear to reflect variations in only air temperature (e.g., PMV estimates) or moisture, indoor enthalpy appears to reflect variations in temperature and RH in the most balanced manner. A series of mixed effects statistical models suggest that higher values of indoor enthalpy appear to be associated with improved cognitive test scores and warm sensations, and warm sensations appear to be associated with improved cognitive test scores. The collective results posit new considerations for the importance of indoor moisture with respect to occupant outcomes and how commonly used modelling approaches may not reflect this. Additional research that incorporates diverse populations, varied built environments, and causal methods could help further our understanding of the effects of air temperature and moisture on occupant outcomes in varied built environment settings.

## 1. Introduction

Indoor thermal conditions have a prominent influence on occupant health, performance, and satisfaction [1-3], and buildings consume substantial amounts of energy to maintain comfortable thermal conditions that support occupants and their activities. Commercial and residential buildings in the United States of America are estimated to consume and emit approximately 40 % of primary energy and greenhouse gas emissions, respectively [4,5], with space conditioning typically being the largest end use, accounting for anywhere from approximately 30–50 % of all end use consumptions [6]. Accordingly, the energy used for space conditioning should achieve the desired goal of providing thermally comfortable indoor spaces that support building occupants and their activities. However, much of the underlying research that informs current building space conditioning practices is based on the Predicted Mean Vote (PMV) model or indoor temperature, which have both been shown to have limited utility in relating indoor thermal conditions to occupant health outcomes.

The PMV model [7,8] is the most recognized thermal comfort model [9] and is used globally [10-12], as it has been incorporated into various standards used in building design and operation for ensuring human thermal comfort [13,14]. The PMV model incorporates four indoor environmental variables (dry-bulb temperature; relative humidity, RH; mean radiant temperature, MRT; and air speed) with two individual thermal variables (clothing insulation and metabolic rate) to predict the mean thermal sensation (not preference) of a building occupant population [8,15]. Although the PMV model has been used pervasively in practice [10] since its creation in 1970 [8,16], there is growing evidence that it has low predictive accuracy when applied to more recent and varied built environment settings. For example, one study used the world’s largest thermal comfort field study database to compare PMV model predictions to occupant thermal sensations votes and found that the PMV model had a predictive accuracy of 34 % while a completely random model had a predictive accuracy of 17 % [17]. Although this model performance is low, it is not totally surprising considering more recent research moving towards individual thermal comfort models [18], noting that thermal comfort is a dynamic and highly individual sensation based on changing environmental conditions and individual physiological and psychological states [19-22].

The challenges with the PMV model in predicting thermal comfort are similar to a related stream of indoor thermal research focused on associations with cognitive performance [23,24]. Several earlier studies investigating indoor thermal conditions, thermal sensations, and metrics of cognitive or work performance observed inverted U-shaped relationships between indoor temperature and performance [22,25,23], and also between thermal sensations and performance [22,24,26]. Based on this early body of research, indoor temperatures around 22 °C [23] and slightly cool to neutral thermal sensations (e.g., −0.5 < PMV < 0) [22,24,26] were believed to promote performance. However, more recent studies on this topic challenge these earlier findings. A 2021 meta-analysis gathered objective measures of indoor air temperature and work performance, normalized the data, and assessed multiple models to represent the relationship between temperature and performance, including the model from the work that helped establish the ~ 22 °C-peaking inverted U-shaped relationship [23], multiple regression models, the Maximum Adaptability framework, and various machine learning techniques. None of the model estimates reached statistical significance (p > 0.05) and all had low predictive accuracies (maximum adjusted R^2^ of 0.07), leading the authors to conclude that none of the models are suitable for accurate predictions and that other factors beyond just temperature are likely important to consider when assessing the relationship between thermal conditions and performance [27].

The collective findings from both streams of research on thermal conditions with respect to a) thermal comfort and b) cognitive performance, arrive at a similar conclusion: established models aimed at predicting these two outcomes have low predictive accuracy, and therefore utility, when applied to modern and varied built environment settings. Since these models inform current building design standards, much of today’s building stock could be consuming unnecessary amounts of energy to achieve indoor thermal conditions that promote neither thermal comfort nor cognitive performance. Evidently, there is a need to look beyond PMV and temperature alone and explore alternative thermal variables in association with occupant outcomes to more accurately assess these relationships and better inform building operation.

Accordingly, we investigate this topic through an opportunity to monitor indoor environmental conditions and multiple occupant outcomes among graduate students in university lecture halls. We measure and develop a suite of thermal variables to explore and then examine associations among these thermal variables, participant thermal sensations, and participant cognitive test scores. The goal of this work is to assess how these different thermal variables are associated with thermal sensations and cognitive performance, and if they can help identify a range of indoor thermal conditions that can support both outcomes across a building occupant population. This work does not seek to develop a thermal comfort prediction model or predict individual thermal sensations. Other research shows that thermal comfort and improved cognitive performance appear to vary based on individual and group-level factors, and that individual comfort models show promise for improved predictive accuracy. However, regardless of individual variations in thermal preferences and the ability to predict them, indoor spaces, within zones, are still conditioned to a single set of thermal conditions. Moreover, many of the personal and dynamic factors (e.g., clothing insulation, metabolism, hormonal states) that help predict individual thermal comfort are often unavailable to researchers or building operators, making individual thermal comfort difficult to provide, let alone predict, in typical building settings. Accordingly, our approach in this study uses measurements of temperature and RH, as they are more easily obtainable and accessible in research and practice, and examines associations on a building occupant population level to help inform practical building operation considerations. Overall, this work introduces new considerations for characterizing and understanding indoor thermal conditions with respect to occupant health outcomes in a practical manner for both research and practice.

## 2. Methods

### 2.1. Study design

This topic is investigated through a prospective, observational, longitudinal cohort study over the 2022–2023 academic year at a Boston-based university. As a result of the COVID-19 pandemic, multiple schools within the university installed real-time, continuous indoor environmental quality monitors in classrooms as an ongoing risk management strategy. Since these monitors provide a continuous assessment of indoor environmental quality, recruiting graduate students attending class in these monitored spaces was an effective approach to investigate this topic. Participation primarily involved completing short thermal perception surveys and cognitive tests after regularly scheduled classes using a smartphone-based research application that had been used in similar studies [28-31]. This outcome data was later paired with the measured class conditions for further analysis and association modeling. All study protocols were General Data Regulation-compliant, reviewed and approved by the Institutional Review Board at the Harvard T.H. Chan School of Public Health, and reviewed and approved by the academic offices of participating students.

### 2.2. Study population and setting

Participants were recruited from two schools at the University via advertisements approved and administered by each school. Study recruitment began at the first school in October 2022 and at the second school in January 2023, due to different approval timelines, and remained open until the end of the study in May 2023. To be eligible for study participation, a series of eligibility criteria had to be met, which included: be a full-time student in one of the participating academic programs; regularly attend class in person; be between eighteen and 65 years of age; have a smartphone compatible with the study application; not be colorblind, in order to complete one of the cognitive tests; not be a current smoker, as current smoking is associated with worse cognitive performance [32] and could heavily influence cognitive test scores; and not have a medical condition or take any medication that could impact one’s thermoregulation. Study participants regularly attended eighty- or ninety-minute classes in one of the two buildings, depending on their graduate program. As it usually takes thirty minutes to acclimatize to a new thermal state [33,34], these class periods provide acclimatization time and additional exposure time. The number and timing of classes varied among participants based on their program and course schedules. Classrooms at each school were similar in terms of design and operation. Both schools included high efficiency particle filtration (MERV 13 or higher) and demand controlled ventilation, with target rates beyond code minimums that were introduced as a risk management strategy in response to COVID-19.

### 2.3. Exposure assessment: indoor thermal conditions and air quality

Indoor thermal conditions are the primary exposure of interest. Indoor dry-bulb temperature (referred to herein as “temperature”) and RH are the two thermal variables measured by the indoor environmental monitors in the classrooms. The monitors also measure carbon dioxide (CO_2_), total volatile organic compounds (TVOCs), fine particulate matter (PM_2.5_), pressure, sound, and a light metric. Of all measured parameters, only temperature and RH are incorporated as exposure variables, and CO_2_ as a confounder; all other measured variables were deemed irrelevant to this analysis as confounders or predictors due to theoretical rationale or a lack of variation or magnitude in the measured values. For example, while past work has shown olfactory perceptions to be intertwined with thermal perceptions [35-37], the class average TVOC and PM_2.5_ concentrations were all low and comparable among classrooms (class average TVOC mean ± SD of 342 ± 198 ppb; all class average PM_2.5_ concentrations < 2 μg/m^3^) and are therefore unlikely to predict or confound the results. However, sensor accuracy (described further in Appendix A) limits the confidence in these measured values and their utility, further supporting the decision to not utilize these measurements in the formal analysis of this work. Furthermore, although lighting and acoustics are associated with cognitive performance, these variables are not associated with thermal conditions in these classrooms (e.g., dimming the lights does not impact temperature) and therefore do not confound the association of interest in this work.

According to the environmental monitor’s manufacturer specifications, measurements are made at the following accuracies: ± 0.5 °C at 25 °C for temperature, ± 3 % for RH, ± 50 ppm or 3 % for CO_2_ (when temperature is between 15 and 35 °C and RH is between 0 % and 80 %). A full table of sensor specifications is provided in Table S1 of Appendix A. Although quality assurance and quality control testing could not be conducted since monitors were already installed by facility teams for operational purposes, all classroom conditions remained within the range of recommended operating conditions of 4–40 °C and less than 85 % RH, so the measurements should be within the manufacturer reported accuracies. Moreover, all sensors within the monitor came pre-calibrated and were given at least seven days of startup time (which is required for the CO_2_ sensor due to an ongoing self-calibration method) before any measurements were included in the study dataset. All three variables were measured and logged at a five-minute measurement frequency over the study period. One monitor logged only temperature and RH from March 8 through April 4, 2023 due to low battery power. Over this duration, the missing CO_2_ values for this one room were replaced with the CO_2_ measurements from the monitor with the strongest correlation (examined with regressions and Spearman Rank correlations to account for potential bias), which appropriately, was the adjacent classroom. With the missing data replaced, class average values of all three variables were then calculated for each class period that corresponded to a participant test or survey response. Only six class periods in the final analysis dataset were impacted by the missing CO_2_ data.

The class average temperature and RH values were retained for direct analysis and to calculate a suite of thermal variables for further exploration. The suite of eleven thermal variables includes indoor variables, consisting of: temperature (°C), RH (%), the partial pressure of water vapor (Pwv, units of kPa), humidity ratio (HR, units of kg moisture/kg dry air), enthalpy (kJ/kg), dewpoint temperature (°C), heat index (a metric that reflects the combined sensations of temperature and RH, expressed in °C), and the estimated PMV score (unitless); and variables that contrast indoor conditions to those outdoors, including: temperature difference (°C), enthalpy difference (kJ/kg), and vapor pressure balance (VPB, units of kPa). All variables, except for PMV, were calculated objectively in RStudio using only the class average temperature and RH values, and outdoor average conditions for the indoor-outdoor difference variables. A description of the calculation approach for these thermal variables is provided in Appendix A. PMV estimates were developed using the “comf” package in R [38] with a series of assumptions, including an average metabolic rate of 1.0 (corresponding to the seated desk work of students) [13], an airspeed of 0.1 m/s (which is the default value for indoor spaces with low air speed, as confirmed by physical spot measurements), MRT equaling air temperature (since most classrooms in this study do not have exterior windows and past work has shown MRT to approximate air temperature in these spaces [39]), and a clothing insulation value of 1.3 clo, calculated based on an estimated ensemble appropriate for the New England heating season, which this study spanned [40]. These assumptions are representative of the study conditions and similar to the estimation approach used in other past work [22,41].

### 2.4. Outcome assessment: thermal perceptions and cognitive performance

The two outcomes of interest in this study are thermal perceptions and cognitive performance. Both outcomes were assessed using the smartphone-based research application. Participants received a thermal perception survey, paired with a cognitive test to complete within a limited time after class. Participants were initially given two hours after class to complete the activities, but this was later shortened to twenty minutes to improve the acuteness of outcome responses with respect to class exposures. Supporting this change was the observation that 63 % of completed study activities within the two-hour window were done within the first twenty minutes. Participants were made aware of the time change and always received a notification on their smartphone when study activities became available and ten minutes prior to their expiration. Other efforts were made to promote participation, including gamifying study activities with points and awarding them as incentives on an on-going basis as accrued.

**Fig. 1.**
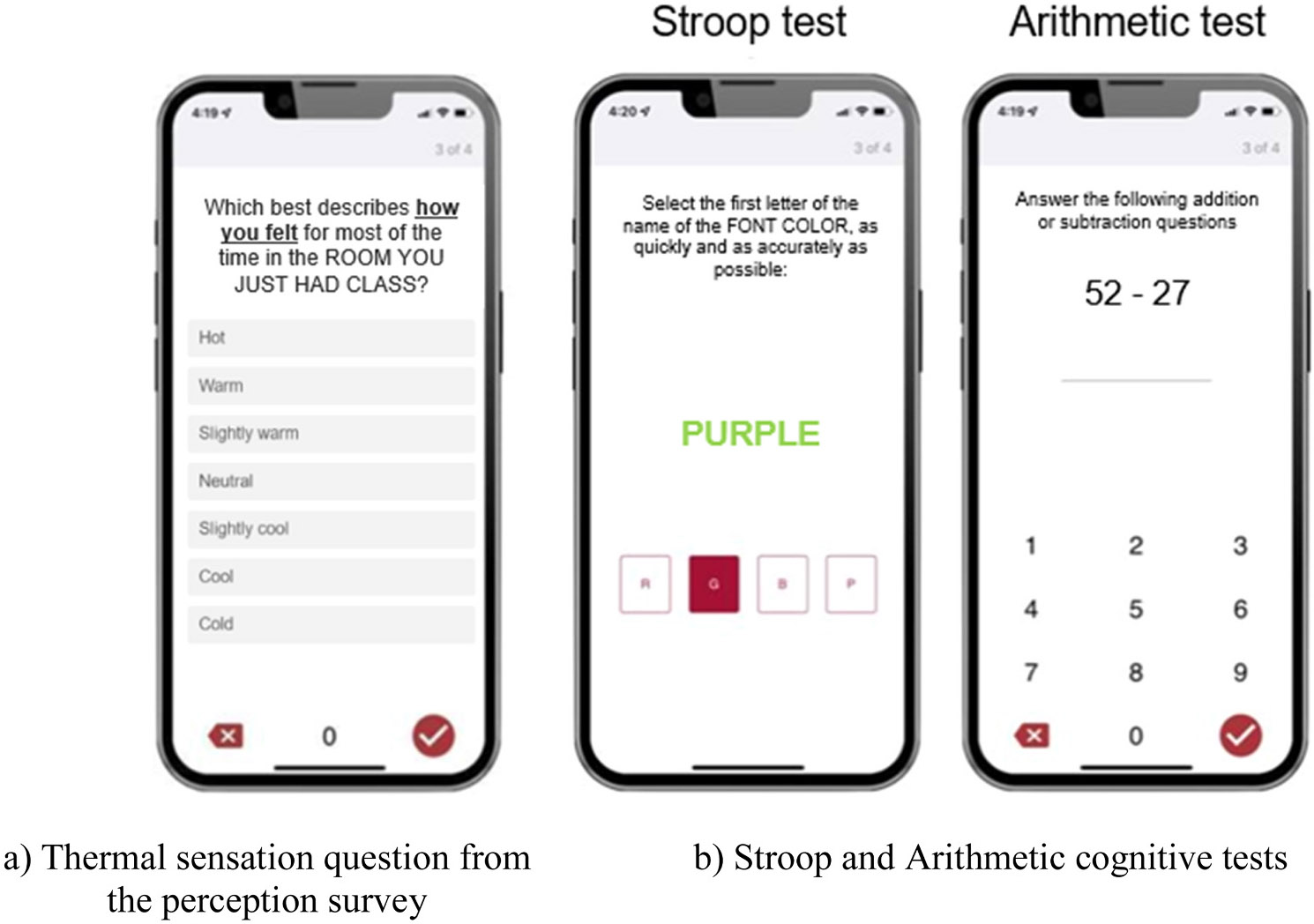
Example of study activities that participants received via the smartphone-based study application.

Thermal perceptions were assessed by administering short thermal surveys. Responses to all core thermal questions were assessed, which informed the decision of focusing our analysis on participant’s thermal sensations. This decision is further supported by recent work that has found the thermal sensation vote to be the most significant thermal comfort predictor [42]. Our survey question asked participants how they felt in the room they just had class in for the majority of time and provided response options in the form of a seven-point Likert scale (i.e., “hot”, “warm”, “slightly warm”, “neutral”, “slightly cool”, “cool”, “cold”, illustrated in Fig. 1) following the American Society of Heating Refrigeration and Air Conditioning Engineers’ thermal sensation scale [19], which is heavily utilized in thermal comfort research [43,44]. Fig. 1a presents this core question as participants received it through the study application with the seven-point Likert scale response option. Due to the frequency of responses per category (most responses being “neutral”, discussed further in the Results section), the more granular responses from the seven-point Likert scale were combined into three categories: “neutral” responses remained in their own category, termed “neutral”; the three responses on the warm side of neutral (i.e., “slightly warm”, “warm”, and “hot”) were combined into the “warm” sensation group; and the three responses on the cool side of neutral (i.e., “slightly cool”, “cool”, and “cold”) were combined into the “cool” sensation group. Herein, this outcome is referred to as “thermal sensation” with the three response options: “warm”, “neutral”, and “cool”.

Cognitive performance was assessed by calculating various metrics from participant responses to validated cognitive performance tests. Two tests were used in this study, including the Stroop Color Word Test (referred to herein as “Stroop”) and the Arithmetic test. The Stroop test assesses selective attention and inhibitory control while the Arithmetic test assesses cognitive speed and working memory. So, while we calculate similar metrics from the test responses, the two tests address different cognitive processes. The Stroop test in this study consists of twenty individual Stroop trials where in each trial, there is a text prompt and the participant must select the color of the font and not the written color. Fig. 1b illustrates an example Stroop trial where the correct answer is “green”. A Stroop trial can be congruent (where the font color matches the written color), incongruent (where the font color does not match the written color), and neutral (where the written text is not a color, but “XXXX”). The Arithmetic test is more familiar and consists of ten trials of two-digit addition and subtraction problems. Fig. 1 also provides an example of an Arithmetic trial where the correct answer is “25”.

Responses to all trials in a single test (twenty for Stroop and ten for Arithmetic) were used to calculate various cognitive test scores. Accuracy and speed are the common dependent variables of these tests, so we calculate throughput, the number of correct responses per unit time (n correct / minute), and total response time (milliseconds) for responses to each test, accordingly. For Stroop responses, we calculate two additional metrics that aim to represent the “Stroop effect”, which refers to reductions in cognitive performance when inhibiting dominant information to select subservient information. In this study, the Stroop effect pertains to selecting the color of the font (subservient information) over the written word (dominant information) and manifests as slowed response times and/or reduced accuracy when completing incongruent trials. There are many metrics that can be calculated to represent the Stroop effect, and much variation in the approach due to how the test was administered and other decisions made by researchers [45,46]. In this study, ratios of incongruent, congruent, and neutral prompts are not equal across all Stroop tests, so we calculate “Stroop incongruent throughput” as the percentage of correct responses per unit time (% correct / minute) and average response time (milliseconds) for incongruent trials within each test. The calculated metrics were cleaned prior to analysis. Any test responses with less than 25 % correct responses or a high response time (one minute and forty seconds for Stroop tests and two minutes and thirty seconds for Arithmetic tests) were excluded as it was likely that a genuine, distraction-free attempt was not made [28,29,47].

### 2.5. Statistical methods

**Fig. 2.**
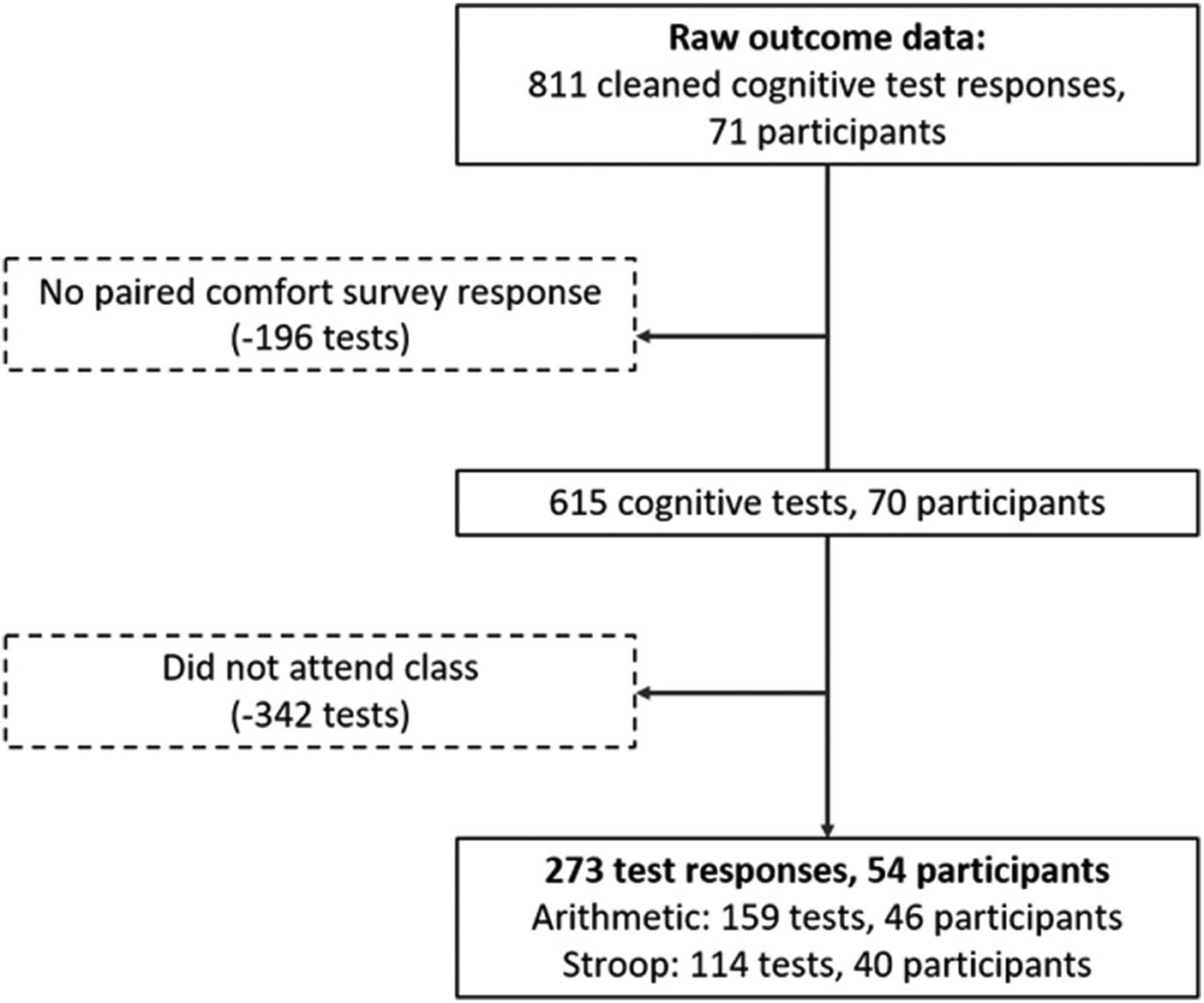
Flowchart showing data inclusion criteria that produced the final analysis dataset.

After scoring and cleaning, calculated cognitive test scores and categorized thermal sensation responses were combined with class average exposure data to create an analysis dataset. Only responses where in-person class attendance was verified (via a survey question) and both the cognitive test and thermal perception survey were completed were included in the final analysis. This resulted in 273 observations from 54 unique participants, as depicted in Fig. 2 below. The 114 Stroop test responses and 159 Arithmetic test responses included in this analysis occurred between November 2022 and April 2023.

Summary statistics, distributions, and Spearman Rank correlation matrices using mean class conditions were first examined to understand the exposure and outcome data. These preliminary findings (primarily three groupings of tight Spearman Rank correlations, discussed further throughout the Results and Discussion sections) motivated additional supporting analyses among the thermal variables. We conducted factor analysis where we began with six factors and followed the Kaiser rule where factors are eliminated when the Sum of Square loadings (Eigen-values) are less than one, which indicates that these factors do not add additional information to the assessment. We also performed principal component analysis to assess linear composites of the variables.

This preliminary work also informed a series of mixed effects statistical models that incorporated the eleven thermal variables (as exposures), six cognitive test scores (as outcomes), and three summarized thermal sensations (as exposures or outcomes) into different exposure-outcome pairings for association modeling. As described earlier, the two primary associations of interest include: 1) the associations between thermal conditions and cognitive test scores, and 2) the associations between thermal conditions and thermal sensations. As a complementary third association, we treated thermal sensations as an exposure and investigated associations with the cognitive test scores. For each of these three associations, we constructed models between each applicable exposure and outcome variable. For example, to investigate the association between thermal conditions and cognitive test scores, a model was constructed for every thermal variable and cognitive test score combination, resulting in 66 models (i.e., eleven thermal variables and six cognitive test scores). For the other investigated associations, we constructed eighteen models exploring the association between thermal sensations and cognitive test scores (three thermal sensation variables and six different test scores), and 33 models exploring the association between thermal conditions and thermal sensation (eleven thermal variables and three thermal sensations).

All models, regardless of exposure or outcome, controlled for class average CO_2_ and school to control for confounding and potential differences between the two schools, respectively. Thermal exposures and CO_2_ were modeled with cubic regression splines to allow for non-linear associations with the outcome of interest in each model. Participant-specific random effects were included to account for correlations among observations from the same individual over time. Beyond these general considerations, additional specifications were made based on the specific outcome.

For models with thermal sensation as the outcome, we constructed a series of binomial regression models for each of the three categorized sensations (i.e., cold, neutral, warm) as binary variables (e.g., cold or not, neutral or not, warm or not). For models with cognitive test scores as the outcomes, we used a linear model with a normal distribution to model each of Stroop throughput, Stroop incongruent throughput, and Arithmetic throughput. Stroop response time, Arithmetic response time, and Stroop average incongruent response time all had to be log-transformed to meet the assumptions of normality and were then assessed using linear models with a normal distribution. Additionally, we controlled for learning effects in all cognitive models by either controlling for the first test response or test number. For Stroop-based models, we controlled for test number since we consistently observed the presence of continued learning effects, based on statistically significant and increasing magnitudes of effect estimates with increasing test number. This decision was also supported by the lower Akaike Information Criterion (AIC) values for models that controlled for test number compared to models that controlled for first test. For Arithmetic-based tests, we consistently observed the opposite: there was no evidence of continued learning effects and the AIC values were lower for models that controlled for first test compared to models that controlled for test number. We controlled for first test in Arithmetic-based models accordingly.

Following base model construction, sensitivity analyses were conducted to further investigate the results. Considering the extent of literature citing perceptions of indoor air quality and thermal conditions to be intertwined [25,35-37,48], we conducted a supporting analysis to assess if air quality issues might be influencing results focused on thermal sensations. Although the CO_2_ concentrations (and TVOC and PM_2.5_ measurements, but not included analysis) were low in this study, compared to other indoor environments [49], we investigated potential correlations and associations between class average CO_2_ concentrations and participant responses to survey questions asking about perceptions of indoor air quality. Responses to survey questions asking participants to rate the quality of indoor air and the presence of any air quality issues were paired with class average CO_2_ concentrations in the same fashion as the analysis dataset. We then constructed boxplots; t-tests and analysis of variance tests, depending on the number of response options; and finally, simple logistic regression models. The logistic regression models incorporated a spline term for class average CO_2_ as the primary exposure variable; controlled for temperature, RH, and school; and included participant-specific random effects. Two models were constructed: one with a binary outcome variable for indoor air quality rated as either “good” or “bad”, and another model using a binary indicator for the presence or absence of air quality issues. Beyond this olfactory investigation, we also ran a series of models that incorporated participant-specific random effects nested within classroom-specific random effects to account for potential correlations among multiple measurements from students in the same classrooms over time. Finally, we ran a series of models with just indoor RH (no paired temperature), outdoor enthalpy, and outdoor dewpoint temperature to assess if the associations we observed with indoor variables (i.e., enthalpy) were indeed distinct or an artifact related to some other variable. All data management, processing, and statistical analysis was conducted in RStudio.

## 3. Results

### 3.1. Participant characteristics

54 unique participants contributed data to the final analysis dataset. Participant characteristics are presented in Table 1 for 53 of these participants as one participant did not complete the background surveys. Most participants in this study are female (~74 %), in their twenties (mean age of 26 ± 3.5 years), and White or Asian.

### 3.2. Measured classroom conditions and calculated thermal variables

A summary of the suite of calculated class average thermal variables and accompanying class average CO_2_ concentrations used in the statistical analysis are presented in Table 2 below. Class average indoor temperatures range from 20 to 26 °C, with a tight interquartile range of 23–24 °C. On average, mean class CO_2_ concentrations are low (i.e., mean and 75th percentile values are 710 and 775 ppm, respectively) compared to concentrations observed in past investigations [49] and inferred from the temporal evolution of minimum standards, which much of today’s building stock achieves or falls below [50-53]. The classrooms in this study had increased outdoor air ventilation rates as an infection risk management strategy, which agrees with these low CO_2_ values. Overall, class average RH is low (i.e., mean and 75th percentile values of 25 % and 28 %, respectively) compared to the range of 30–60 % [54] that is recommended for human health and comfort considerations (in addition to other built environment considerations, such as material durability). The two metrics, heat index and PMV, focused on characterizing human thermal perceptions reveal that the heat index reflects a slightly lower temperature compared to dry-bulb air temperature in this study and that all estimated PMV values are negative, suggesting that on average, this study population would have felt cool according to the PMV estimates.

**Fig. 3.**
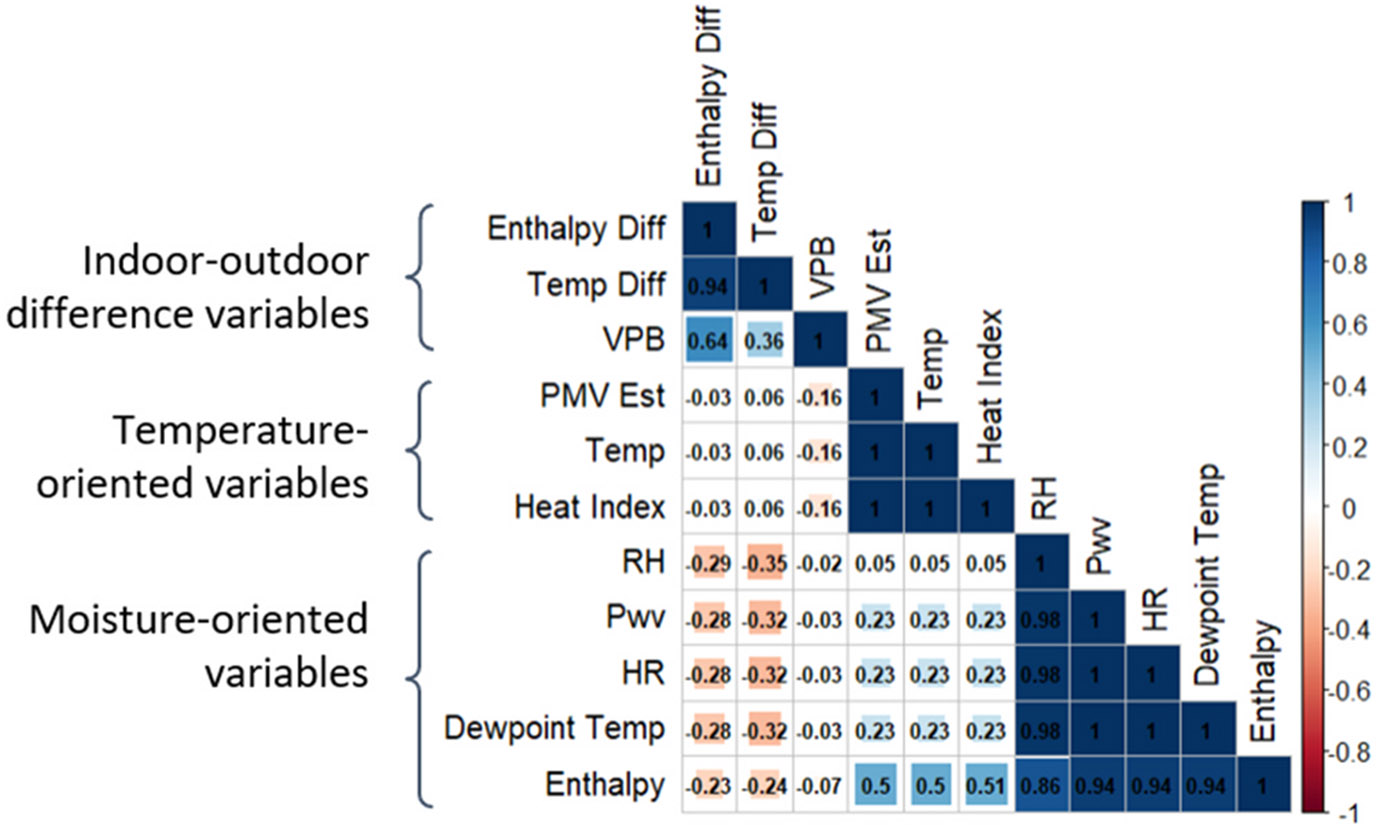
Spearman rank correlation coefficients showing three distinct groupings among class average thermal exposure variables.

Moving on to the assessment of correlation and variability among thermal variables, the Spearman-rank correlation coefficients are presented in Fig. 3, which show three groupings of very tight correlations around temperature, RH, and indoor-outdoor differences. Motivated by the intensity of these clusters, we conducted factor analysis, which yielded three factors that account for 96 % of the overall variance across all eleven variables, and whose factor loadings follow the three groups defined by the Spearman Rank correlations. These results were further supported by agreeing results from the principal component analysis. These complementary analyses confirm the three observed groupings of thermal variables, which we define and refer to herein as: 1) temperature-oriented variables, consisting of temperature, heat index, and estimated PMV; 2) moisture-oriented variables, consisting of RH, Pwv, humidity ratio, dewpoint temperature, and enthalpy; and 3) indoor-outdoor difference variables, consisting of the temperature difference, enthalpy difference, and VPB. We observe perfect 1:1 Spearman Rank correlations among all variables within the temperature-oriented group and low correlations with variables in the moisture-oriented group, suggesting that the heat index and PMV estimates strongly correlate with air temperature and not moisture in this study. Similarly, for the moisture-oriented group, we observe strong positive Spearman Rank correlations among all variables within this group and low correlations with variables in the temperature-oriented group. Enthalpy is somewhat distinct as it has the lowest, but still strong, correlation with RH within this group (correlation coefficient of 0.86) and the strongest correlation with temperature in this group (correlation coefficient of 0.51), suggesting that enthalpy might correlate with both air temperature and moisture in a more balanced and comprehensive manner than the other temperature and moisture-oriented variables. Finally, we observe the lowest correlation coefficients within the indoor-outdoor difference group, which combined with higher residual variance for the factor loadings in this group from our factor analysis, suggests higher variability within this group. Furthermore, indoor-outdoor difference variables show overall low negative correlations with the temperature and moisture-oriented variables, suggesting these variables provide distinct information compared to the other two groups focused on indoor conditions.

### 3.3. Thermal sensations and cognitive test scores

**Fig. 4.**
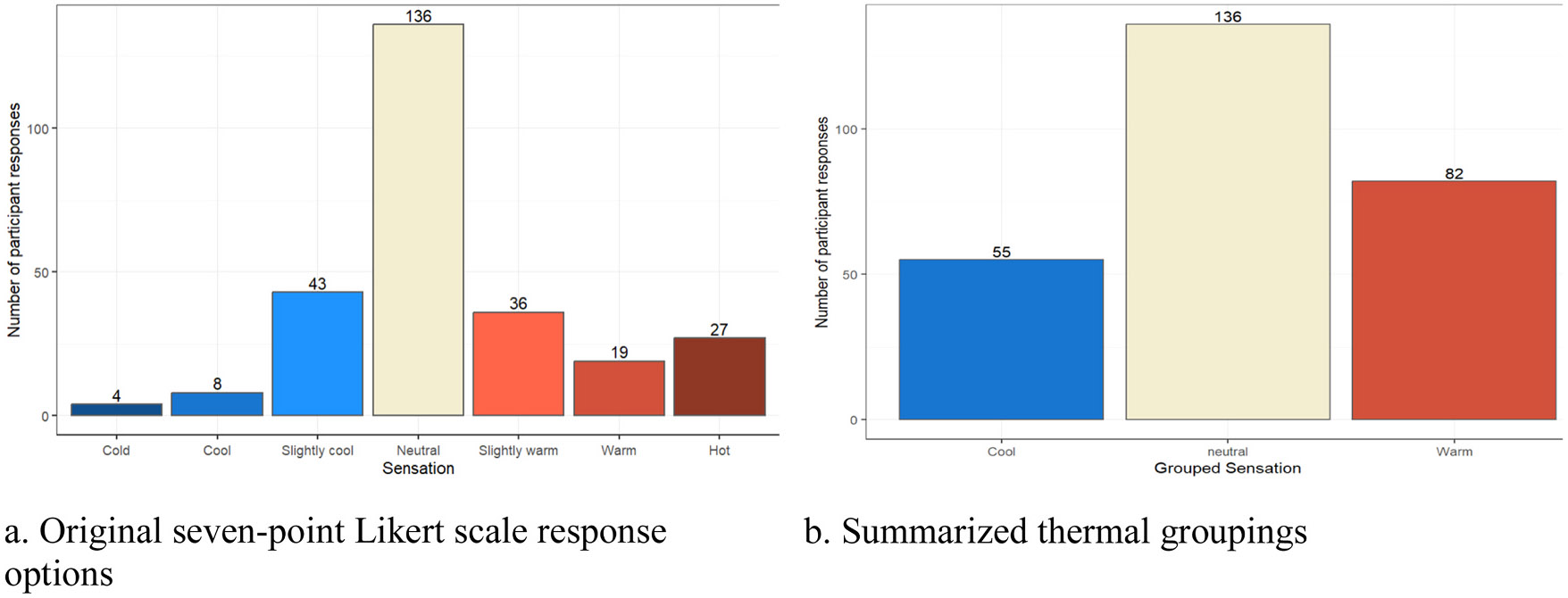
Summary of participant’s thermal sensations in the rooms they just had class.

Participant responses to the core thermal sensation question are presented in Fig. 4 below, with the original seven-point Likert scale responses summarized on the left (Fig. 4a) and the three categorized sensations summarized on the right (Fig. 4b). As shown, 50 % of thermal sensation responses were neutral, 30 % were some sensation of warm, and 20 % were some sensation of cool, which counters the PMV estimates that suggested most participants would feel a cool sensation. The small number of responses in the individual, more granular cold and warm sensation options in the seven-point Likert scale (Fig. 4a) motivated the three sensation groupings shown in Fig. 4b, as previously described, for the association modeling.

Calculated cognitive test scores indicate that on average, one Stroop trial takes 1.2 seconds ( ± 0.4) to complete and that 50 ( ± 19) correct Stroop trials can typically completed per minute (i.e., average Stroop throughput). Focusing on incongruent Stroop trials only, to represent the Stroop effect, we observe an average response time of 1.3 seconds ( ± 0.5) per trial and a Stroop incongruent throughput metric of 4.1 % / minute ( ± 1.7). Each arithmetic trial takes approximately 5.1 seconds ( ± 1.8) to complete and approximately 12 correct trials ( ± 4) are completed each minute, on average (i.e., arithmetic throughput).

### 3.4. Modeled associations

Since we observed such tight correlations among thermal variables within each of the three groups, it is not surprising that the modeled associations are similar among thermal variables within the same group. Accordingly, and for brevity, we discuss the overall pattern of association observed for each of the three thermal groups and then further illustrate these patterns by presenting more detailed model results for one representative thermal variable from the temperature and moisture-oriented groups. We discuss temperature (paired with RH as a separate independent variable) from the temperature-oriented group and enthalpy from the moisture-oriented group based on practical and theoretical rationale. Specifically, the use of temperature and RH as separate independent variables is common practice in association modeling and epidemiology, general built environment research, and real-world building assessments, and accordingly, we highlight temperature and RH to emphasize the implications of using this approach. We select enthalpy from the moisture-oriented group due to the findings from the thermal exposure assessment that it potentially correlates with the temperature and moisture of air in a distinct and more balanced manner and could be a useful metric for relating indoor thermal conditions to occupant outcomes. We do not present detailed results for the indoor-outdoor difference group, as these variables showed little evidence of meaningful, consistent, or statistically significant associations. The results are presented with respect to the range of exposures and outcomes included in this analysis only. We refer to “statistically significant evidence” as results reaching the 0.05 alpha level (**), and “mixed evidence” as a combination/group of results reaching and not reaching the 0.05 (**) and 0.1 (*) alpha levels.

#### 3.4.1. Associations between thermal variables and cognitive performance

**Fig. 5.**
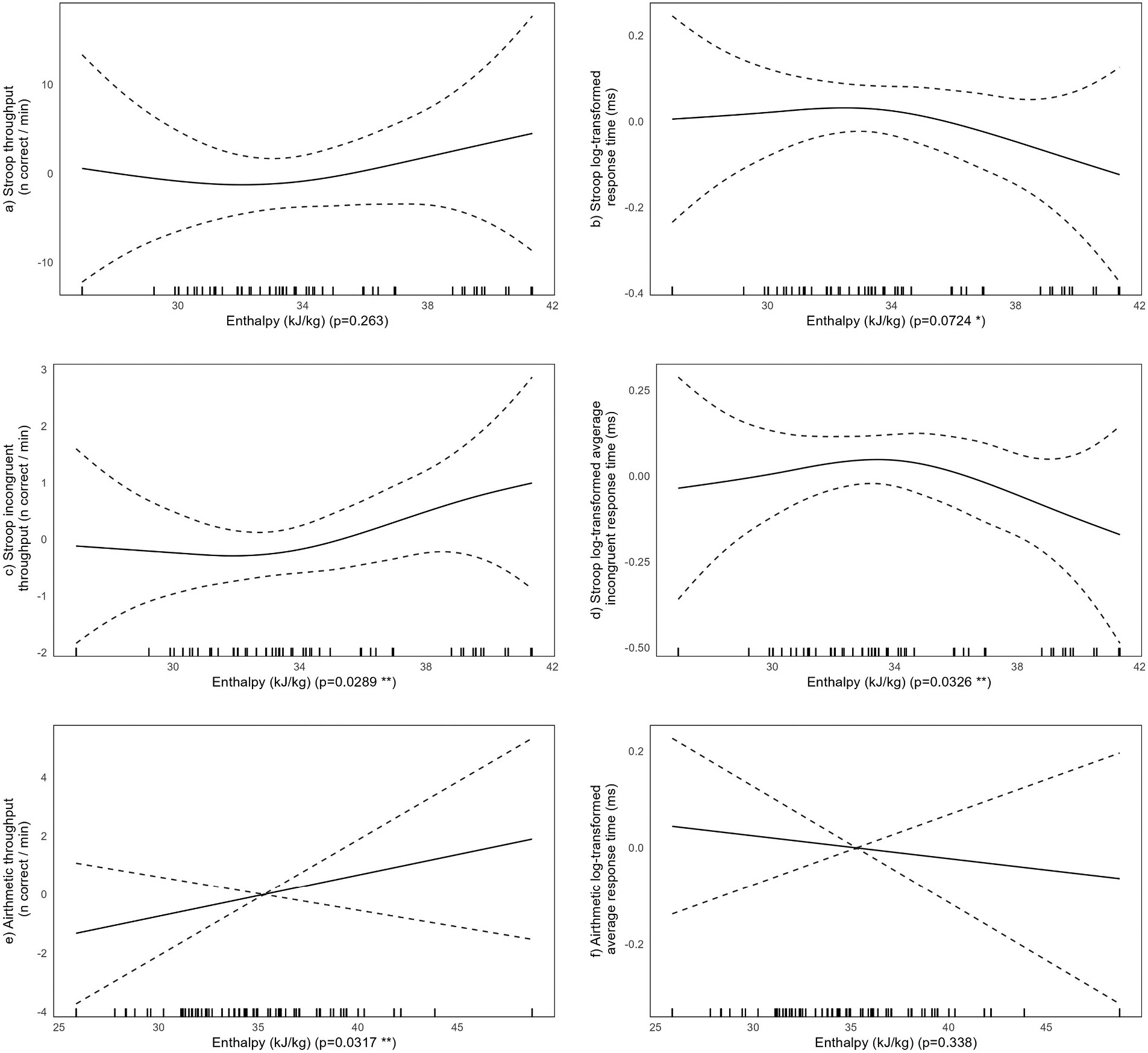
Plotted spline terms from adjusted mixed effects models showing associations between indoor enthalpy and each of the six cognitive scores. Associations appear nonlinear for Stroop scores and linear for arithmetic scores. For all scores, better cognitive scores (higher throughput or lower response times) are observed at the higher range of enthalpy compared to the lower range. Models are adjusted for class average enthalpy and CO_2_, school, and learning effects, and include participant-specific random effects.

We first examined model estimates for associations between thermal conditions and cognitive test scores. Beginning with the moisture-oriented group, spline terms showing the modeled associations between indoor enthalpy and the six cognitive test scores are presented in Fig. 5 below. The modeled associations suggest linear associations between enthalpy and arithmetic test scores (Fig. 5e-f), where increases in indoor enthalpy are associated with increases in throughput and decreases in response time. Accordingly, we re-ran the arithmetic models with linear terms for enthalpy and found that a 10 kJ/kg increase in indoor enthalpy is associated with roughly three additional correct arithmetic responses per minute (p = 0.032 **) and a 5 % faster response time (p = 0.34). There is evidence of nonlinear associations between indoor enthalpy and the four Stroop scores (Fig. 5a-d); overall, we see suggestive evidence that higher enthalpy, over the 26–49 kJ/kg range examined in this analysis, is associated with better Stroop scores as we observe higher throughput scores and lower response times at the higher compared to lower enthalpy range. The model estimates are mixed in terms of statistical significance, as detailed for each spline term in Fig. 5 below.

Moving on to the temperature-oriented variables, model estimates suggest that higher values of temperature-oriented variables are linearly associated with improved cognitive performance for all cognitive scores except Stroop throughput where we observe a nonlinear association with temperatures around 24 °C being associated with the highest Stroop throughput scores for all three thermal variables in the temperature-oriented group. The modeled associations are inconsistent for RH. While in some instances, increases in RH appear to be associated with improved cognitive scores, there are many instances where the modeled spline terms suggest no association with cognitive scores. Statistically, the results are mixed for temperature and none of the accompanying RH splines reach any level of statistical significance. The set of models that includes temperature and RH as separate thermal exposure variables suggests a stronger and more consistent association between temperature and cognitive test scores compared to RH and cognitive test scores. We present representative plotted spline terms for temperature and RH from this set of models in Appendix A. For the indoor-outdoor difference group, we do not find evidence of a consistent association with all six cognitive test scores for each of the exposure variables in this group, and accordingly, no meaningful pattern of an association for the group itself.

#### 3.4.2. Associations between thermal variables and thermal sensation

Next, we examined modelled associations between the eleven thermal variables and three binary thermal sensation variables (warm or not, neutral or not, cool or not). For temperature-oriented variables, we observe an inverted U-shaped association, peaking around 23–24 °C, with feeling neutral as opposed to not, as shown in Appendix A. Not surprisingly, we found higher values of both temperature and moisture-oriented variables to be associated with warm sensations, and lower values to be associated with cool sensations. We observe the strongest statistical evidence of associations for models predicting warm sensations, mixed evidence for models predicting cold sensations, and no statistically significant evidence for models predicting neutral sensations. Once again, we do not observe evidence of a consistent association between indoor-outdoor difference variables and thermal sensation. Sample spline terms are presented in Appendix A.

#### 3.4.3. Associations between thermal sensation and cognitive performance

Finally, as a complementary assessment, we examine the modeled associations between thermal sensations (neutral or not, warm or not, cool or not) and cognitive performance (i.e., the six cognitive test scores). We find neither directionally consistent nor statistically significant evidence that feeling neutral as opposed to not is associated with cognitive test scores. Instead, we find directionally consistent evidence, with mixed statistical significance, that feeling warm as opposed to not is associated with improved correct responses per time, and lower response times. Conversely, feeling cool as opposed to not is associated with worse cognitive test scores (both correct responses per time and response time), but none of these effect estimates reach statistical significance at the 0.05 or 0.1 alpha levels. These model estimates are presented in Table S2 Appendix A.

#### 3.4.4. Sensitivity analyses

Collectively, we found no evidence of a correlation or association between CO_2_ and air quality perceptions, as boxplot distributions were similar, *t*-test and analysis of variance results showed no significant differences between groups, and the regression models showed no evidence of an association (spline terms were nearly horizontal lines). The regression models also show little evidence of an association between temperature and RH with indoor air quality perceptions. This suggests that participants did not typically note issues with the indoor air quality and that there does not appear to be olfactory interference with thermal perceptions. This furthers our confidence in this study’s findings that indoor enthalpy appears to be associated with thermal sensations and cognitive performance, separately from any olfactory issues. We observed no differences between model estimates with and without classroom as a nested random effect. Finally, the series of models with just indoor RH (no paired temperature as a separate variable), outdoor enthalpy, and outdoor dewpoint temperature did not yield consistent or statistically significant evidence of associations, further strengthening our observations with indoor temperature and moisture-oriented variables, despite our small dataset and limited study scenario.

## 4. Discussion

In this work, we set out to explore a suite and thermal variables and their utility in estimating associations with occupant outcomes, including: 1) thermal conditions and cognitive performance, 2) thermal conditions and thermal sensation, and, as a complement, 3) thermal sensations and cognitive performance. In the process, we observed interesting relationships among thermal variables themselves, how they relate to cognitive performance and thermal sensations, and implications for our treatment and characterizations of thermal conditions.

### 4.1. Thermal variable groups: implications for quantifying indoor thermal conditions

The first key finding of this work pertains to the correlation and variability assessment among thermal variables and the implications for the quantification of indoor thermal conditions. The Spearman Rank correlations, factor analysis, and principal component analysis show that by constructing the suite of eleven thermal variables, we’re actually measuring three things, which reflect indoor temperature, indoor moisture, and indoor-outdoor differences. While PMV or heat index are often calculated to better reflect the thermal sensations experienced by individuals [29], our findings show they are essentially representing variations in indoor temperature in a different way in our study setting. This is not necessarily surprising for heat index, given its common use in outdoor settings at temperature and RH conditions much higher than those observed in this study, and its stepwise pattern with low sensitivity to changes in the temperature and moisture range reflective of typical indoor conditions, as in our study. For PMV, our use of static estimates for MRT, air speed, clothing insulation and metabolic rate will contribute to its perfect correlation with temperature. With more dynamic, specific, and accurate information for these variables, the PMV estimates and correlation with temperature would likely vary [55]; however since this information is difficult to obtain in research and practice, our assumption-based estimation approach is not uncommon, and the estimation of PMV in similar scenarios could also yield PMV estimates that reflect variations in indoor temperature. While temperature, PMV, and heat index are commonly used quantifications of thermal conditions, it appears that in many indoor settings, these variables could reflect variations in indoor temperature and not moisture, despite PMV and heat index including RH in their calculations. Although indoor enthalpy correlates more strongly with indoor RH, its more balanced correlations suggest that it might better represent variations in both temperature and moisture, which could be important for quantifying combined thermal conditions and relating them to occupant health.

### 4.2. Modeled associations

The implications from the exposure correlation and variability assessment are observed in the modelled associations. The set of models that use PMV and heat index as the thermal exposure variable reflect the temperature effect estimates, and not RH, from the model that incorporates temperature and RH as separate independent variables. For this latter modelling approach, we observed directionally consistent and statistically stronger evidence of an association between temperature and the outcomes compared to RH. Temperature and RH are commonly modelled as separate independent variables [56], but our findings suggest that this approach might emphasize the association of temperature and not the potential importance of indoor moisture or the combined effect of temperature and moisture with respect to the occupant outcomes examined. Indeed, past work has shown that considering temperature and RH separately can be inadequate, as the two variables can move in different directions, further supported by the low Spearman correlation (0.05) observed in this work, yet work together to influence occupant health [57]. Evidently, the temperature-oriented variables, either heat index, estimated PMV, or temperature with RH as a complementary independent variable, appear to emphasize the role of temperature and not moisture.

Moving on to the moisture-oriented variables, we also observe strong positive correlations among variables within this group and consistent patterns of modelled associations, with enthalpy as a somewhat distinct variable. Although the analysis dataset is small, the collective evidence suggests that enthalpy might incorporate both air temperature and moisture in a more representative manner that is distinct from the other variables in this analysis, which can be useful for better representing and assessing the combined effects of temperature and RH on occupant health. Finally, the indoor-outdoor difference variables required additional outdoor weather data to calculate but did not provide consistent evidence of meaningful associations with the outcomes of interest. This aligns with them being less proximate/related to the indoor exposures experienced by participants in their classrooms and observations from existing work where others have commented on the low utility of indoor-outdoor differences for predicting occupant comfort [58].

Beyond these considerations related to the thermal exposure variables themselves, we observed interesting patterns of modeled associations that raise questions related to the influence of indoor air temperature and moisture on human occupants. This study occurred over the heating season in the greater Boston area and the tight range of mean indoor temperatures (range of 20–26 °C, IQR of 23–24 °C) are within a range deemed “comfortable” [13] and typical of most work environments [27,59]. However, mean indoor moisture was low (range of 12–46 %, IQR of 18–28 %) relative to the 30–60 % RH range that is recommended for human comfort and health, among other building considerations [54]. Accordingly, the tighter range of air temperatures and larger range of RH measurements suggests a tighter range of heat and a larger range of moisture in the air. While the temperature-oriented variables emphasize the heat component, and most moisture-oriented variables emphasize the moisture component, enthalpy incorporated both in the most balanced manner. Enthalpy is a measure of the total energy content of the air, including the sensible heat of dry air and the latent heat of moisture in the air [60,61]. Indoor enthalpy correlates more closely with the moisture-oriented variables, which, combined with the tight range of temperatures and larger range of low indoor RH, suggests that in this study, increases in air moisture are likely responsible for the increases in enthalpy, which is observed to be associated with improved cognitive performance and warm sensations. This suggests that participants felt warmer and performed better with higher indoor air moisture, over the tight range of acceptable indoor temperatures and a larger, but also small, range of indoor RH observed in this study. More broadly, these findings suggest that indoor air moisture might be more influential to these outcomes than recent attention has devoted, since much past work has focused on indoor temperatures (or the other temperature-oriented variables, PMV and heat index) and/or a modeling approach (e.g., temperature and RH as separate variables) that potentially underemphasizes the importance of moisture. Collectively, the findings from this investigation suggest that indoor moisture, particularly when expressed with its paired temperature as a conjugate metric and not as a separate, independent variable, as is the case with temperature and RH as separate variables, might be more relevant to cognitive performance than past attention has given. These findings suggest that enthalpy might be a more complete representation of both the heat and moisture content of air with respect to these occupant outcomes, and particularly important when trying to examine the indoor conditions that support occupants and their activities.

Comparison of the present findings to past work is challenging due to the limited amount of published research that examines indoor enthalpy or thermal parameters beyond temperature and RH in the context of associations with cognitive performance and/or thermal sensations. A recent systematic literature review on the topic examined sixty studies that met inclusion criteria and found that 90 % of studies utilized temperature; some works also assessed RH, CO_2_, and other metrics of light and sound; and none of the reviewed studies assessed indoor enthalpy, dewpoint temperature, humidity ratio, vapor pressure, or indoor-outdoor differences [56]. One recent study examined associations between heat index and Stroop metrics and found nonlinear results [29], while other occupational work has used wetbulb globe temperature meter readings to find an association between heat stress and impaired Stroop test scores [62]. A few previous studies examined indoor enthalpy and occupant satisfaction, but with respect to perceptions of indoor air quality and found that higher indoor enthalpy, beyond the range measured in our study, was associated with poorer perceptions of indoor air quality [35-37]. A more recent study examined indoor enthalpy and occupant satisfaction (both with thermal and air quality conditions) under hot and humid conditions and found at high enthalpies, beyond those in the present study (>55 kJ/kg), dissatisfaction with air indoor air quality is greater than dissatisfaction with thermal conditions and that the Fanger models (percent dissatisfied, using PMV) underpredicted the level of dissatisfaction [58]. These authors note other motivating works where air acceptability decreased with increasing enthalpy, but above the range in our study [63,64]. Our hypothesis that increases in indoor moisture over the measured range in our study could be driving increases in indoor enthalpy, which are associated with improved performance, is supported by past work that has found increases in indoor moisture to be associated with reduced symptoms of irritation [65], lower stress and improved sleep [66], reduced absenteeism [67], and reduced virus transmission [68,69].

### 4.3. Mechanisms of thermal exposures on cognitive performance and thermal sensation

Although not focused on enthalpy, Harriman [70] looks beyond indoor temperature and RH as individual parameters and discusses dewpoint temperature with respect to thermal comfort. He states that human comfort is driven by differences in dewpoint temperatures between the skin surface and surrounding ambient air. A bigger difference between the two indicates greater drying potential, which is beneficial when you want to release heat to cool off (i.e., during the cooling season), but detrimental when you want to conserve heat and not “dry out” to stay warm and comfortable (i.e., during the heating season) [70]. Relatedly, others have examined skin wettedness and also noted its importance to thermal comfort. One study examined participants during exercise and recovery and found skin wettedness to be more important to thermal behavior than core temperature [71]. Other studies have focused on more accurately representing skin wettedness and evaporative heat loss in thermal comfort models, yielding substantial improvements in model predictive accuracy. One study utilized the standard effective temperature model, with advanced human thermoregulation, to better represent skin wettedness and heat loss via evaporation at the skin surface into the PMV model. They then validated this model with the American Society of Heating, Refrigeration, and Air Conditioning Engineer’s Global Thermal Comfort Database II and found the predictive accuracy to improve from 32 % for the original PMV model to 64 % for their modified model for a variety of building types and settings [72]. These authors also assessed related model modifications by other investigators who did not perform their own validations. They found that similar efforts, such as a PMV adaptation aimed at more accurately accounting for evaporative heat loss by basal sweating [73], improved PMV prediction accuracy by 49 %. Evidently, skin wettedness and the associated evaporative heat loss with surrounding air shows promising importance for thermal comfort. In our study over the heating season, we observe mean class dewpoint temperatures ranging from −8.4–12 °C, with an IQR of −1.6–5 °C and a mean of 1.6 °C ± 4 °C. Harriman [70] notes that a higher dewpoint means the air feels muggier and at lower dewpoints, symptoms, such as dry eyes and skin, start to emerge. He recommends maintaining a dewpoint between roughly −1.1 and 4.4 °C during the heating season to help promote thermal comfort, while being mindful of the energy expenditure associated with this space conditioning. Most class mean dewpoint temperatures in our study fall within this range, and most participants voted feeling neutral, supporting Harriman’s recommendation. However, some dewpoint temperatures in our study fall below this range, and beyond comfort, based on our association modeling, it appears that higher levels of indoor moisture could be beneficial to cognitive performance. Our findings with enthalpy could be related to these suggested thermoregulation mechanisms between skin wettedness and air via moisture exchange, especially given the high correlation between indoor enthalpy and dewpoint temperature (Spearman Rank correlation coefficient of 0.94). While we hypothesize skin-air moisture-based interactions as part of the underlying biological mechanisms of our modeled associations between moisture-oriented variables and occupant outcomes, it is likely that other mechanisms are at play with respect to indoor thermal conditions and thermal sensations and cognitive performance. Past studies have noted improved cognitive performance slightly beyond neutral sensations, which many attribute to mental arousal from stimulation caused by changes in brain temperatures [74,75]. Furthermore, the improved cognitive performance we observed with higher levels of indoor moisture over the range in our study could be due to the aforementioned occurrences of reduced symptoms of irritation [65], lower stress and improved sleep [66], reduced absenteeism [67], and reduced virus transmission [68,69].

### 4.4. Limitations and next steps

There are many inherent limitations in this work that must be considered when interpreting these results. First, our study population and scenario limits generalizability. Our study consists primarily of White and Asian female participants in their mid-twenties with high levels of education. Past work has found females to prefer and perform better under warmer conditions compared to males [20,76-78] and that older individuals are more susceptible to the effects of thermal conditions [79], which means that the findings from this study might not apply to populations of more diverse sex and age. Furthermore, the indoor spaces in this study are located in the Boston area and the measurement period included in analysis pertains to the heating season only. Accordingly, the low values of measured RH in our study and apparent benefit of higher indoor enthalpy (greater energy related to air temperature and particularly moisture) pertain to the low and tight range of thermal conditions observed in our study and might not translate to other times of year and studies in cooling-dominant climates. Indeed, past work occurring in cooling-dominated settings found slightly cool sensations and/or lower indoor moisture to improve cognitive performance [22,24,26,80-82], even among a similar population of female graduate students, age 16–23, but in Saudi Arabia [80]. Similarly, it has been found that occupants prefer and adapt to indoor thermal conditions differently based on their regional background and typical climate [80,83], which again, implies that these results would differ based on population and location.

Moreover, although we assess multiple thermal variables, we acknowledge that our assessment is limited to quantifications of air temperature and moisture, which is not thermally comprehensive. Although we made a series of investigative field measurements of surface temperatures and airspeed, many limitations (i.e., accuracy and measurement range of the devices, no knowledge of participant locations to calculate MRT) prevented the use of these measurements in our analysis. While most rooms are interior with no windows to the outdoors and airspeeds were low, our assumptions for MRT and airspeed to calculate PMV are reasonable, but not fully accurate, along with our static assumptions for clothing insulation and metabolic rate, which are highly variable in practice [57]. Future work could also assess these parameters for a more comprehensive assessment of indoor thermal conditions since the surface temperature scans did reveal some variation in indoor surface temperature from interior heat sources. Nevertheless, our results are still useful for research and practice where these involved field measurements are not possible and simple measurements of temperature and RH are available through in-room monitors and the building management system.

With respect to our outcome measures, there is the possibility of recall error as participants answered thermal surveys after class, but within a set time limit. Furthermore, the cognitive tests used enable the objective calculation of cognitive scores, but do not reflect the range of cognitive processes and work performance performed in all environments, such as creative thinking [29,30,84] and more involved strategy [85]. The effect of thermal conditions has been reported to vary based on task type and complexity, with many studies finding no association between temperature and simple tasks, but an association between temperature and complex tasks [86,87]. This suggests that the effect of thermal conditions on work and performance could vary based on the type of work being performed.

Moving on to our statistical approach, although the correlation and variability analysis of thermal variables yielded interesting results, the methods used do not imply causation and the examination of enthalpy is based on the variability assessment and theoretical rationale, not a formal statistical selection method. The Spearman Rank correlation assesses the strength and direction of monotonic relationships and does not address the magnitude of differences or causality. Similarly, although our statistical models control for many potential confounders while also providing the flexibility to explore non-linear associations, these are not causal methods, and so interpreting the results is limited to comparing and contrasting the modeled associations and hypothesizing potential differences and/or explanations.

Despite these limitations, the study design and statistical approach instill confidence in our findings, which provide new considerations for the quantification of air temperature and moisture with respect to occupant health. Collectively, the statistical findings and theoretical explanations in the literature suggest that indoor moisture may be more important to these occupant outcomes than previous attention has been given [88,89], and that the commonly used approach of investigating these outcomes with temperature-oriented variables might not elucidate this importance. Our findings with enthalpy at lower values than previously examined [63,64] highlight this importance and present an approach to better incorporate indoor moisture and temperature towards improving our understanding of the indoor thermal environment and its impact on occupants. Since enthalpy is straightforward and scalable to calculate with simple temperature and RH measurements, it has high utility for future studies that investigate how the thermal indoor environment influences occupant outcomes.

Future work would ideally build upon this approach to further understand how indoor moisture, and its quantification, influence occupant outcomes. Future investigations would ideally study a more diverse range of participants, built environment settings and indoor conditions, and occupant outcomes (including different types of cognitive processes and physiologic outcomes) beyond those examined in this study to better understand how these associations might vary across built environment settings. Moreover, future studies would ideally obtain larger datasets to allow for the use of causal methods that could enable statistical selection and help identify causal effects of thermal variables individually, interactively, and collectively. Cumulatively, these efforts could help to better understand the role of indoor moisture and the levels of indoor moisture, along with temperature and other environmental variables, that can support a multitude of occupant health outcomes and activities. This is important to prevent ill-health observed at both the lower and higher bounds of indoor moisture [90,91] and to inform the space conditioning processes [92] and entailed energy requirements [93,94] that can achieve these indoor conditions.

## 5. Conclusion

This prospective, observational, longitudinal study provides new insights surrounding the thermal variables we use to characterize the built environment and how they relate to occupant outcomes. We observe that indoor thermal variables cluster into three groups related to indoor temperature, indoor moisture, and indoor-outdoor differences and each of these groups exhibits distinct patterns of associations with cognitive test scores and thermal sensations. These findings posit important considerations for built environment research and practice, in settings similar to those in this work, including: 1) the eleven thermal variables examined reflect variations in either indoor temperature, indoor moisture, or indoor-outdoor differences; 2) pervasive modelling approaches using PMV estimates or temperature and RH as separate independent variables appear to emphasize variations in indoor air temperature and not moisture; 3) indoor moisture and a quantitative representation of it with temperature, as is the case with enthalpy, might be more important to occupant health outcomes than previously considered; and 4) higher values of indoor enthalpy and warm sensations in this study, likely driven by increases in indoor air moisture, could promote cognitive performance during the heating season. The collective findings suggest an importance of indoor moisture that requires further attention and understanding. These considerations should be explored beyond the limited scope of this study to better understand the role of indoor moisture, representative indoor thermal characterizations, and how these findings might vary with different study populations and built environment settings. Ideally, the collective results could inform new approaches for representing indoor thermal conditions with respect to occupant health for research and practice.

## Supplementary Material

1

Supplementary data associated with this article can be found in the online version at doi:10.1016/j.indenv.2025.100098.

## Figures and Tables

**Table 1:** Participant characteristics for 53 of 54 participants included in analysis^a^.

Characteristic	Overall (N = 53)^b^
**Sex Assigned at Birth**	
Female	39 (73.6 %)
Male	12 (22.6 %)
Prefer not to say	2 (3.8 %)
**Age**	
Mean (SD)	26.2 (3.48)
Median [Min, Max]	26.0 [21.0, 42.0]
**Hispanic, Latino, or Spanish Origin**	
No: not of Hispanic, Latino/a/x, or Spanish origin	45 (84.9 %)
Yes: another Hispanic, Latino/a/x, or Spanish origin	5 (9.4 %)
Yes: Mexican, Mexican American, Chicano/a/x	2 (3.8 %)
Yes: Puerto Rican	1 (1.9 %)
**Race**	
Asian or Asian American	17 (32.1 %)
Black or African American	6 (11.3 %)
Some other race	4 (7.5 %)
White or Caucasian	24 (45.3 %)
White or Caucasian or Native Hawaiian or Other Pacific Islander	1 (1.9 %)
White or Caucasian or Some other race	1 (1.9 %)
**US Origin**	
No	26 (49.1 %)
Yes	27 (50.9 %)

a One of the 54 participants included in analysis did not complete the baseline survey and is therefore not included in the above table of 53 out of 54 participants.

b Quantities presented are counts with percentage of total in brackets, unless otherwise specified with square brackets and a description in the characteristic row.

**Table 2:** Summary statistics for class average indoor conditions included in analysis (n = 273).

Variable	Mean	SD	CV	Min	p25	p50	p75	p95	Max
Temperature (°C)	23	1.2	0.051	20	23	23	24	25	26
RH (%)	25	6.8	0.28	12	19	25	28	36	46
CO_2_ (ppm)	707	207	0.29	471	562	625	771	1176	1309
Heat Index (°C)	22	1.2	0.055	19	22	22	23	24	25
PMV	−1.2	0.43	−0.35	−2.4	−1.4	−1.2	−0.96	−0.58	−0.21
Enthalpy (kJ/kg)	35	3.7	0.11	26	32	34	37	41	49
Pwv (kPa)	0.71	0.2	0.29	0.33	0.54	0.69	0.88	1.1	1.4
Humidity Ratio (kg water/kg dry air)	0.0044	0.0013	0.29	0.002	0.0034	0.0043	0.0054	0.0066	0.0087
Dewpoint (°C)	1.6	4	2.5	−8.4	−1.6	1.7	5	7.8	12
Temperature Difference (°C)	18	5.8	0.33	5.8	12	17	22	26	27
Enthalpy Difference (kJ/kg)	19	8	0.43	4.6	12	19	25	33	42
VPB (kPa)	0.062	0.22	3.6	−0.37	−0.061	0.026	0.16	0.41	0.88
