# Supplementary material for: New considerations for representing moisture in indoor thermal conditions: Associations between enthalpy, cognitive performance, and thermal sensations
Source: Indoor Environ. Author manuscript; Available in PMC 2025 Jul 27. (PMC12290924; doi:10.1016/j.indenv.2025.100098)
Supplement: 1 [file NIHMS2090459-supplement-1.docx]

# **APPENDIX A for:**

# **New considerations for REpresenting moisture in indoor thermal conditions: associations between enthalpy, cognitive performance, and thermal sensations**

This appendix provides additional supporting figures and tables for the three associations examined in this work. Details of the sensor performance characteristics are presented, followed by a description of how the thermal variables were calculated. Additional details from the mixed effects statistical models are presented next. Associations between indoor thermal conditions and cognitive test scores are described by presenting spline terms for temperature and relative humidity for each of the six cognitive tests from the models incorporating both temperature and relative humidity as separate independent thermal variables. Next, model estimates for the association between indoor thermal conditions and thermal sensation are presented, along with sample spline terms from these models. Finally, section three presents effect estimates for the association between thermal sensations and cognitive test scores.

**1) Environmental Sensor Specifications**

*Table S1: Specifications for sensors included in the indoor environmental monitors used in this study.*

| **Parameter** | **Sensor type** | **Range** | **Accuracy** |
| --- | --- | --- | --- |
| Temperature | Not specified | 4-40 °C | ±0.5 °C |
| RH | Not specified | <85% | ±3% |
| CO_2_ | Non-dispersive infrared | 400-5000 ppm | ±50 ppm or ±3% within 15-35 °C and 0-80%RH |
| PM_2.5_ | Laser scattering optical particle counter | 0 ~ 200 ug/m_3_ | ± (5 μg/m³ + 15%). Calibrated with a GRIMM reference instrument using cigarette smoke source. |
| TVOC | Metal oxide gas sensor | Not specified ^a^ | Not specified ^a^ |

**^a^** Manufacturer does not list TVOC sensor performance details and could not provide information when contacted.

**2) Thermal Variable Calculations**

This section outlines the calculation approach taken for each of the thermal variables that was calculated from temperature and RH measurements. Some variables were calculated as a step in a process towards the calculation of another. For these instances, we have grouped thermal variables in accordance with their successive calculations and present the successive steps. For this paper, all calculations were carried out in RStudio.

**Heat Index**

Step 1) First create indoor temp in ° F

temp_F = temp_C*(9/5)+32

Step 2) create RH in decimal percent

rh_d = RH/100

Step 3) now create heat index variable (in ° F)

HeatIndex_F = -42.379 + (2.04901523*temp_F) + (10.14333127* rh_d) - (.22475541* temp_F* rh_d) - (.00683783* temp_F* temp_F) - (.05481717*$rh_d* rh_d) + (.00122874* temp_F* temp_F* rh_d) + (.00085282*temp_F* rh_d* rh_d) - (.00000199* temp_F* temp_F* rh_d* rh_d)

Step 4) Create Heat Index in degree ° C variable (for comparison with units in our study)

HeatIndex_C = (HeatIndex_F-32)*(5/9)

**Dewpoint Temperature:** Both a more “exact” and “approximate” calculation approach was taken and compared. The methods yielded similar results; we utilized the “exact” estimates in our analysis.

Dewpoint_exact = 243.04*(log(RH/100)+((17.625* temp_C)/(243.04+ temp_C)))/(17.625-log(RH/100)-((17.625* temp_C)/(243.04+ temp_C)))

Dewpoint_approximate = temp_C - ((100-RH)/5)

**Partial Pressure of Water Vapor (Pwv), Humidity Ratio (HR), Enthalpy**

Step 1) Calculate dry-bulb temperature in Kelvin (T), given measured temperature in °C

Temp_K = temp_C + 273.15

Step 2) Calculate saturation vapor pressure, Pws (kPa) given temp_K

Pws = (exp(-5800.2206/temp_K + 1.3914993 + -.048640239*temp_K + .000041764768*temp_K^2 + -.000000014452093*temp_K^3 + 6.5459673*log(temp_K)))/1000

Step 3) Calculate partial pressure of water vapor, Pwv (kPa)

Pwv = (RH/100) * Pws

Step 4) Calculate humidity ratio, HR (kg/kg)

HR = 0.62198*(Pwv/(101.325-Pwv))

Step 5) Calculate enthalpy (kJ/kg)

Enthalpy = 1.006*temp_C + HR*(2501+1.86*temp_C)

**Temperature Difference**

Temperature Difference = Indoor Temperature – Outdoor Temperature

**Enthalpy Difference:** Note that outdoor enthalpy was calculated following the steps outlined above, but using outdoor temperature and RH measurements

Enthalpy Difference = Indoor Enthalpy – Outdoor Enthalpy

**Vapor Pressure Balance (VPB)**

VPB = Indoor Pws – Outdoor Pws

## **3) Associations between Thermal Conditions and Cognitive Test Scores**

| a) Stroop throughput  (n correct / min) | 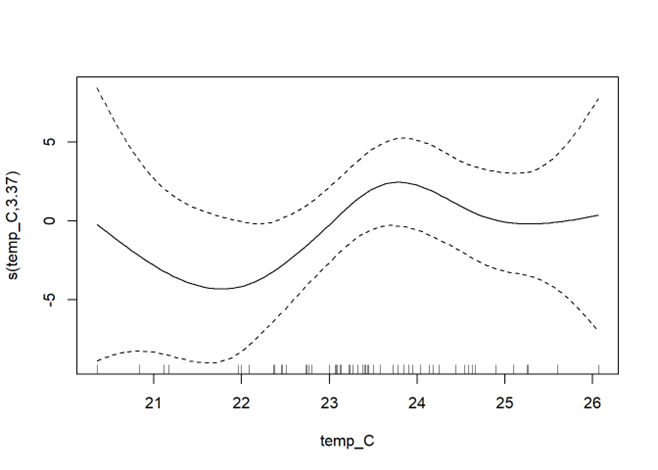 | b) Stroop log-transformed response time (ms) | 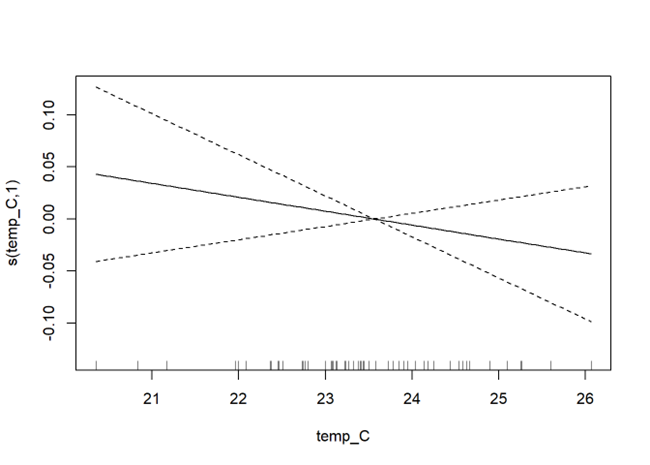 |
| --- | --- | --- | --- |
|  | Temperature (⁰C) (p=0.386) |  | Temperature (⁰C) (p=0.308) |
| c) Stroop incongruent throughput (% correct / min) | 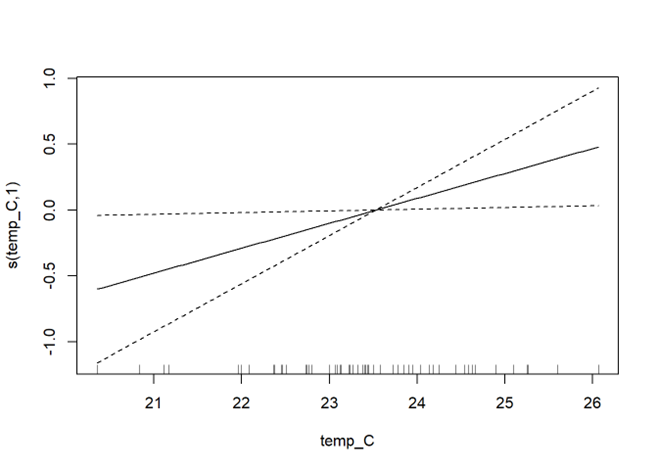 | d) Stroop log-transformed average incongruent response time (ms) | 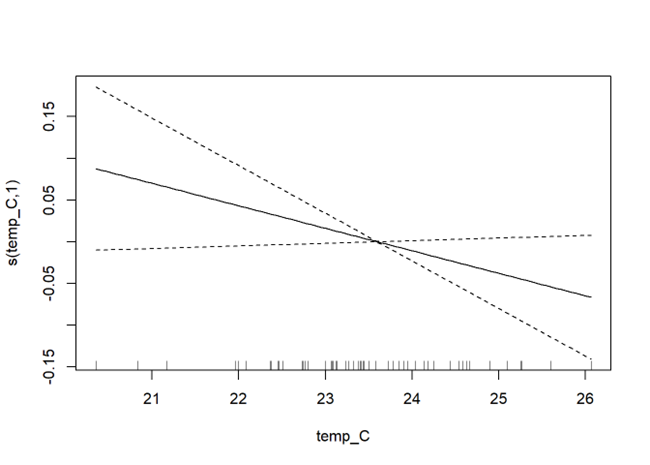 |
|  | Temperature (⁰C) (p=0.0346 **) |  | Temperature (⁰C) (p=0.0767 *) |
| e) Arithmetic throughput  (n correct / min) | 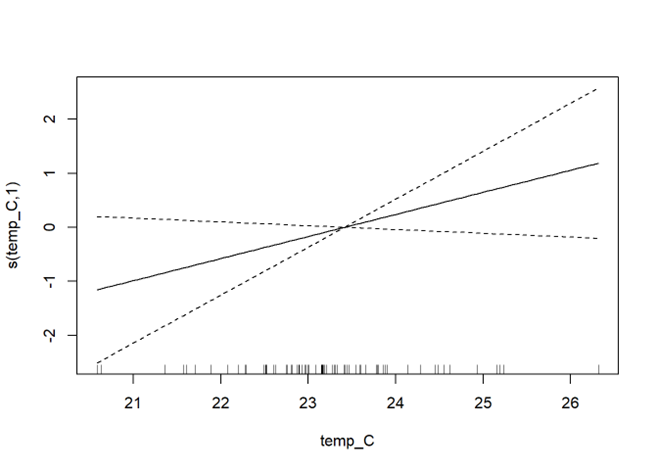 | f) Arithmetic log-transformed response time (ms) | 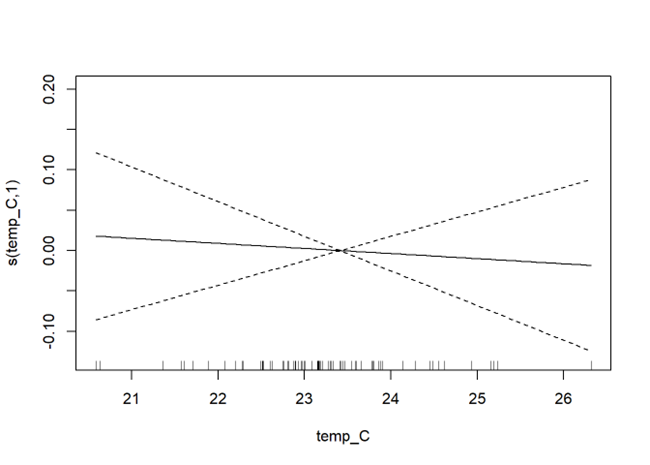 |
|  | Temperature (⁰C) (p=0.0901 *) |  | Temperature (⁰C) (p=0.730) |

Figure S1: Plotted spline terms from adjusted mixed effects models showing the associations between indoor temperature and each of the six cognitive function scores. Models are adjusted for class average temperature, RH and CO_2_, school, and learning effects, and include participant-specific random effects.

| a) Stroop throughput  (n correct / min) | 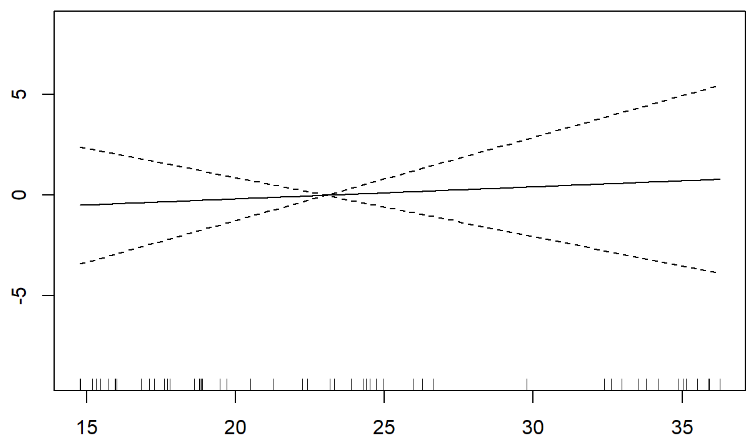 | b) Stroop log-transformed response time (ms) | 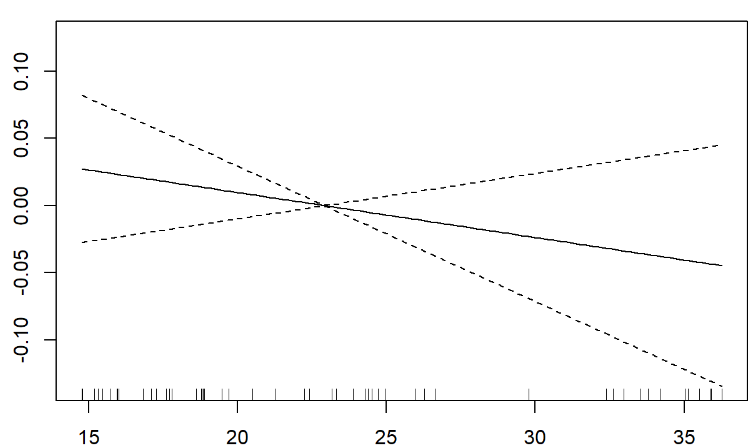 |
| --- | --- | --- | --- |
|  | RH (%) (p=0.735) |  | RH (%) (p=0.322) |
| c) Stroop incongruent thorughput (% correct / min) | 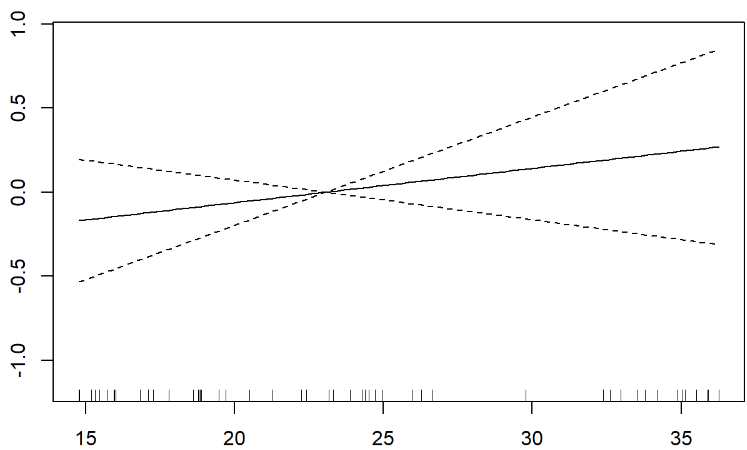 | d) Stroop log-transformed incongruent response time (ms) | 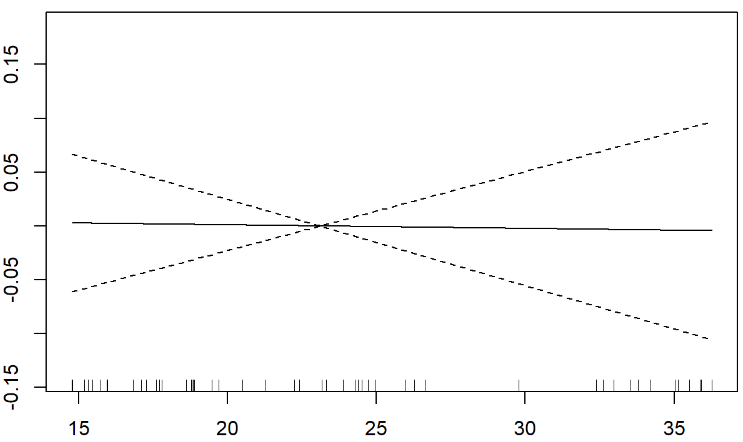 |
|  | RH (%) (p=0.356) |  | RH (%) (p=0.931) |
| e) Arithmetic throughput  (n correct / min) | 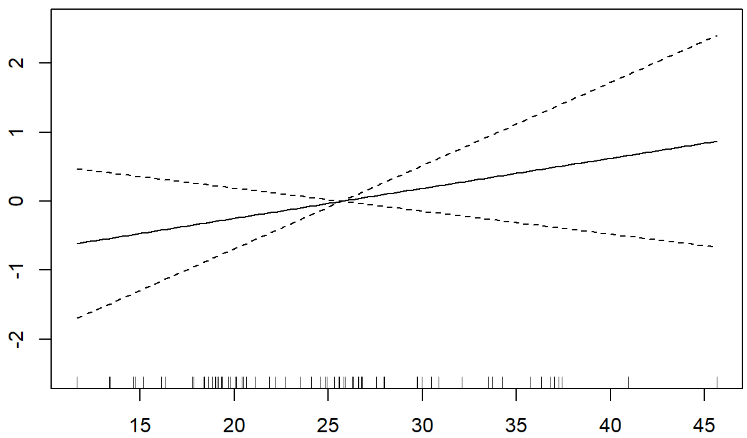 | f) Arithmetic log-transformed response time (ms) | 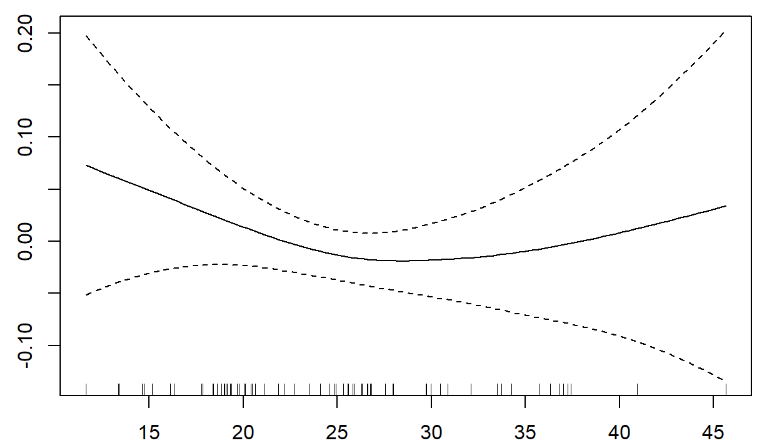 |
|  | RH (%) (p=0.259) |  | RH (%) (p=0.391) |

Figure S2: Plotted spline terms from adjusted mixed effects models showing the associations between indoor RH and each of the six cognitive function scores. Models are adjusted for class average temperature, RH and CO_2_, school, and learning effects, and include participant-specific random effects.

## **4) Associations between Thermal Conditions and Thermal Sensations**

| Binary Sensation  (neutral = 0, not neutral > 0) | 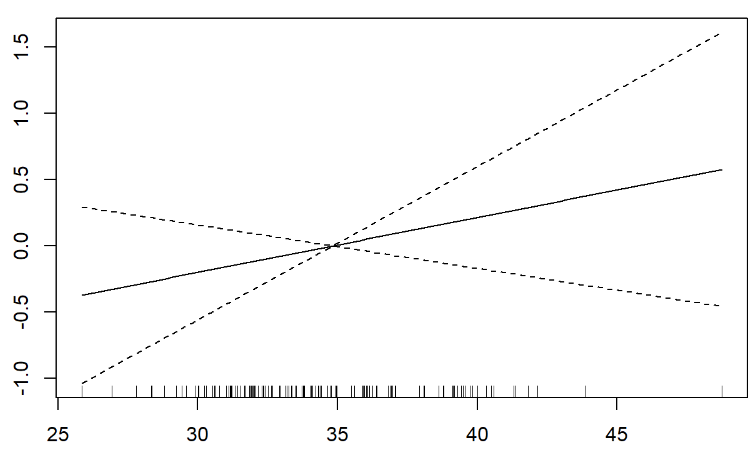 |
| --- | --- |
|  | Enthalpy (kJ/kg) (p=0.265) |

Figure S3: Spline term showing the modeled association between class average enthalpy and neutral sensations. Models are adjusted for class average enthalpy and CO_2_, and school, and include participant-specific random effects.

| Binary Sensation  (neutral = 0, not neutral > 0) | 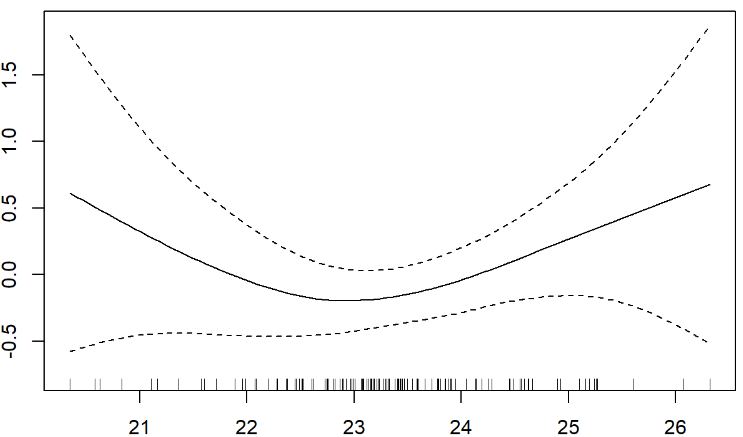 | Binary Sensation  (neutral = 0, not neutral > 0) | 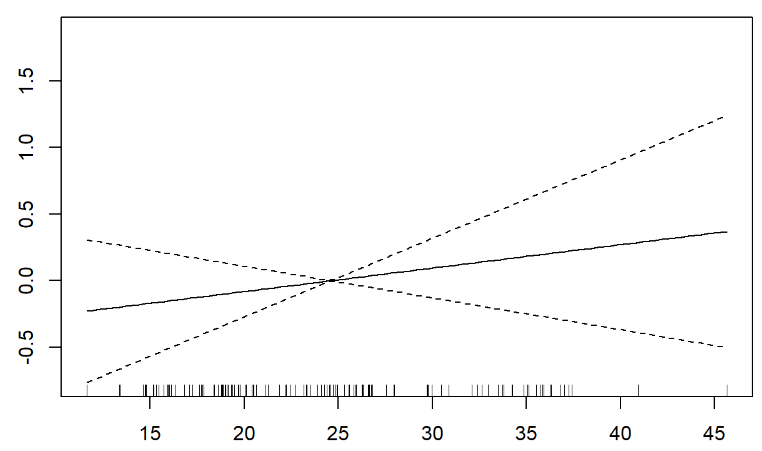 |
| --- | --- | --- | --- |
|  | 1. Temperature (⁰C) (p=0.197) |  | 1. RH (%) (p=0.396) |

Figure S4: Spline term showing the modeled association between class average temperature (a) and RH (b) and neutral sensations. Models are adjusted for class average temperature, RH, and CO_2_, and school, and include participant-specific random effects.

## **5) Associations between Thermal Sensations and Cognitive Test Scores**

Table S2: Effect estimate summary from models exploring the associations between binary thermal sensations and cognitive test scores.

|  | Change in cognitive test score | | |
| --- | --- | --- | --- |
| Cognitive tests, grouped by thermal sensation | Change (95% CI) | p-value | |
| Feeling neutral (as opposed to not) |  |  |  |
| **Stroop (n=113)** |  |  |  |
| Throughput (n correct / minute) | 0.18 (-4.01, 4.37) | 0.93 |  |
| Response time (milliseconds) | 1.02 (0.95, 1.1) | 0.61 |  |
| Incongruent throughput (% correct / minute) | -0.11 (-0.6, 0.38) | 0.65 |  |
| Average incongruent response time (milliseconds) | 1.05 (1, 1.09) | 0.29 |  |
| **Arithmetic (n=159)** |  | 0 |  |
| Throughput (n correct / minute) | -0.1 (-1.2, 1.01) | 0.87 |  |
| Response Time (milliseconds) | 0.99 (0.92, 1.08) | 0.89 |  |
| Feeling warm (as opposed to not) |  |  |  |
| **Stroop (n=113)** |  |  |  |
| Throughput (n correct / minute) | 2.02 (-2.76, 6.8) | 0.41 |  |
| Response time (milliseconds) | 0.94 (0.87, 1.1) | 0.15 |  |
| Incongruent throughput (% correct / minute) | 0.58 (0.03, 1.13) | 0.04 | * |
| Average incongruent response time (milliseconds) | 0.91 (0.87, 0.96) | 0.05 | * |
| **Arithmetic (n=159)** |  |  |  |
| Throughput (n correct / minute) | 0.51 (-0.65, 1.01) | 0.87 |  |
| Response Time (milliseconds) | 0.98 (0.9, 1.07) | 0.65 |  |
| Feeling cold (as opposed to not) |  |  |  |
| **Stroop (n=113)** |  |  |  |
| Throughput (n correct / minute) | -2.64 (-7.81, 2.52) | 0.32 |  |
| Response time (milliseconds) | 1.05 (0.95, 1.14) | 0.34 |  |
| Incongruent throughput (% correct / minute) | -0.51 (-1.08, 0.06) | 0.08 |  |
| Average incongruent response time (milliseconds) | 1.04 (0.99, 1.1) | 0.41 |  |
| **Arithmetic (n=159)** |  |  |  |
| Throughput (n correct / minute) | -0.68 (-2.16, 0.81) | 0.37 |  |
| Response time (milliseconds) | 1.04 (0.93, 1.17) | 0.44 |  |
